# Supplementary material for: Transcriptome and metabolome analysis of crGART, a novel cell model of de novo purine synthesis deficiency: Alterations in CD36 expression and activity
Source: PLoS One. 2021 Jul 20;16(7):e0247227. doi: 10.1371/journal.pone.0247227 (PMC8291708; doi:10.1371/journal.pone.0247227)
Supplement: S1 File — (PDF) [file pone.0247227.s001.pdf]

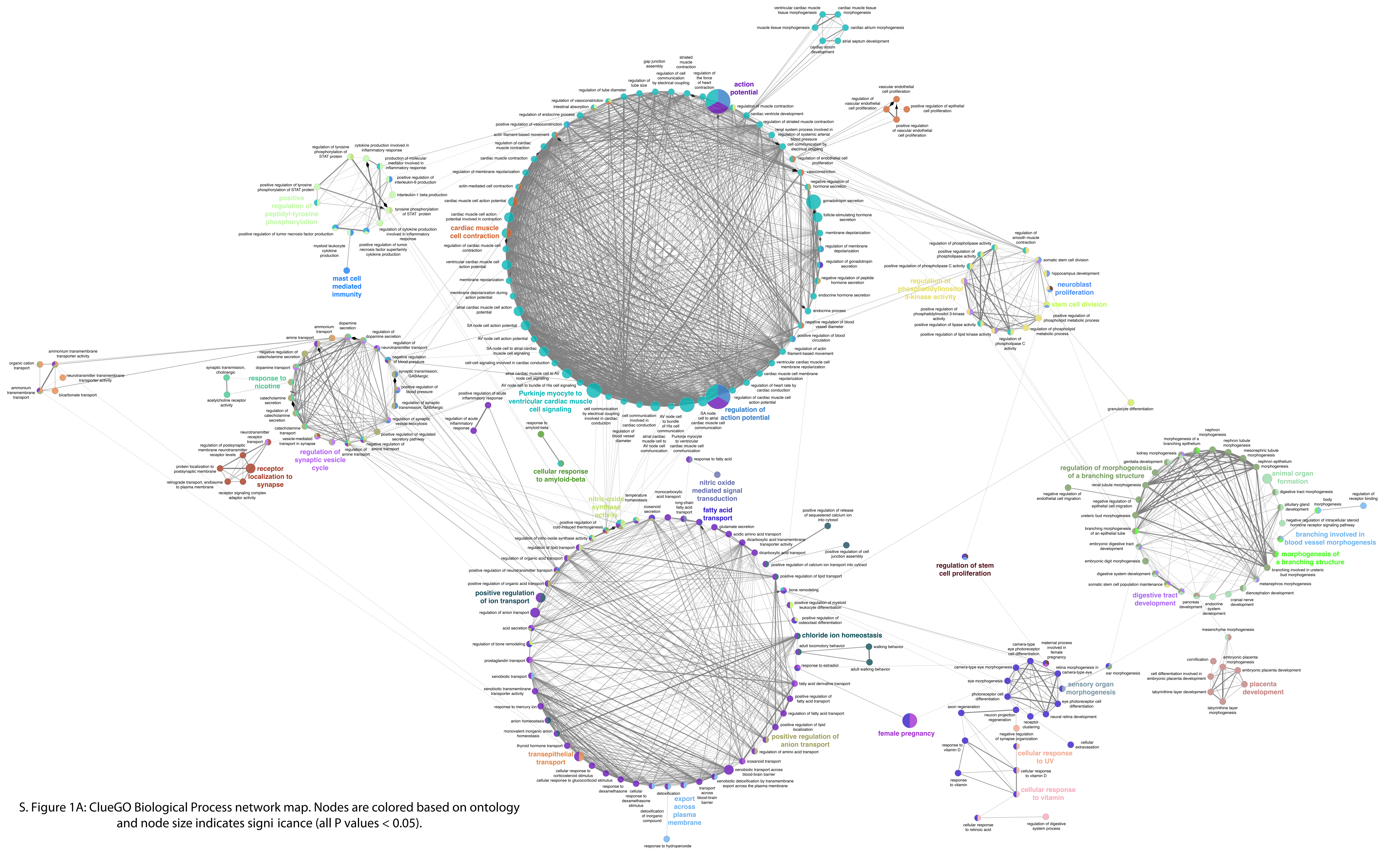

S. Figure 1A: ClueGO Biological Process network map. Nodes are colored based on ontology and node size indicates signi icance (all P values < 0.05).

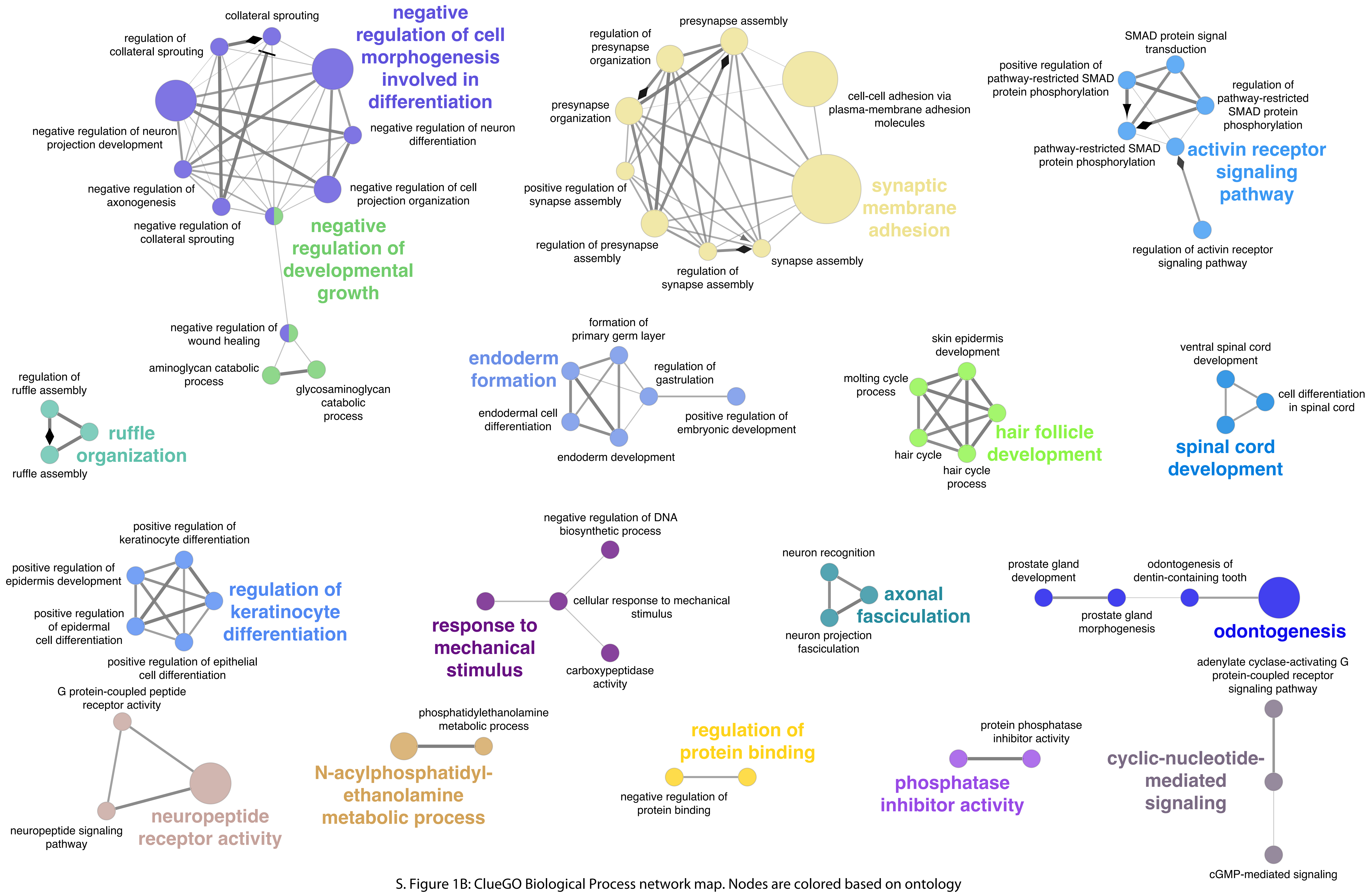

S. Figure 1B: ClueGO Biological Process network map. Nodes are colored based on ontology and node size indicates signi icance (all P values < 0.05).

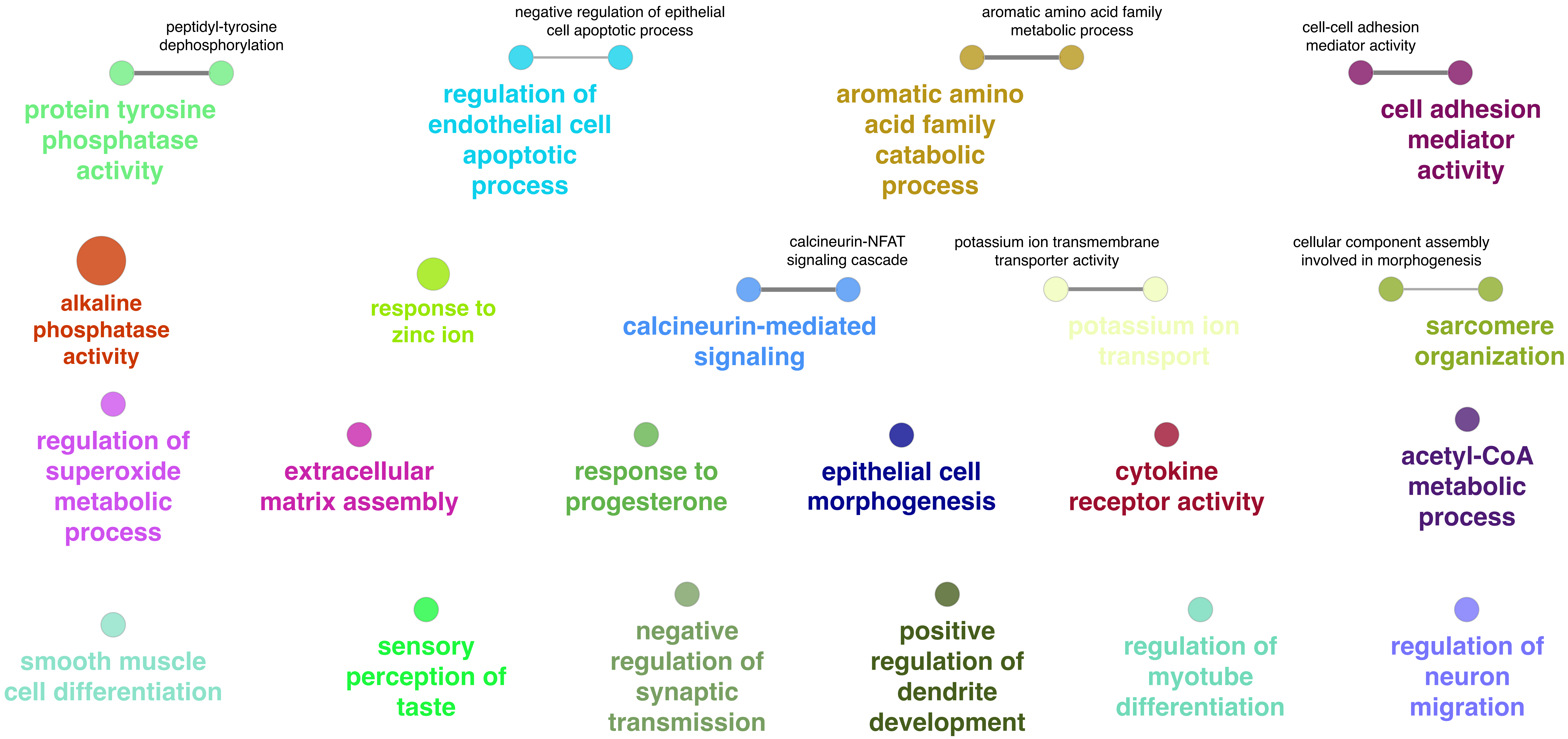

S. Figure 1C: ClueGO Biological Process network map. Nodes are colored based on ontology and node size indicates signi icance (all P values < 0.05).

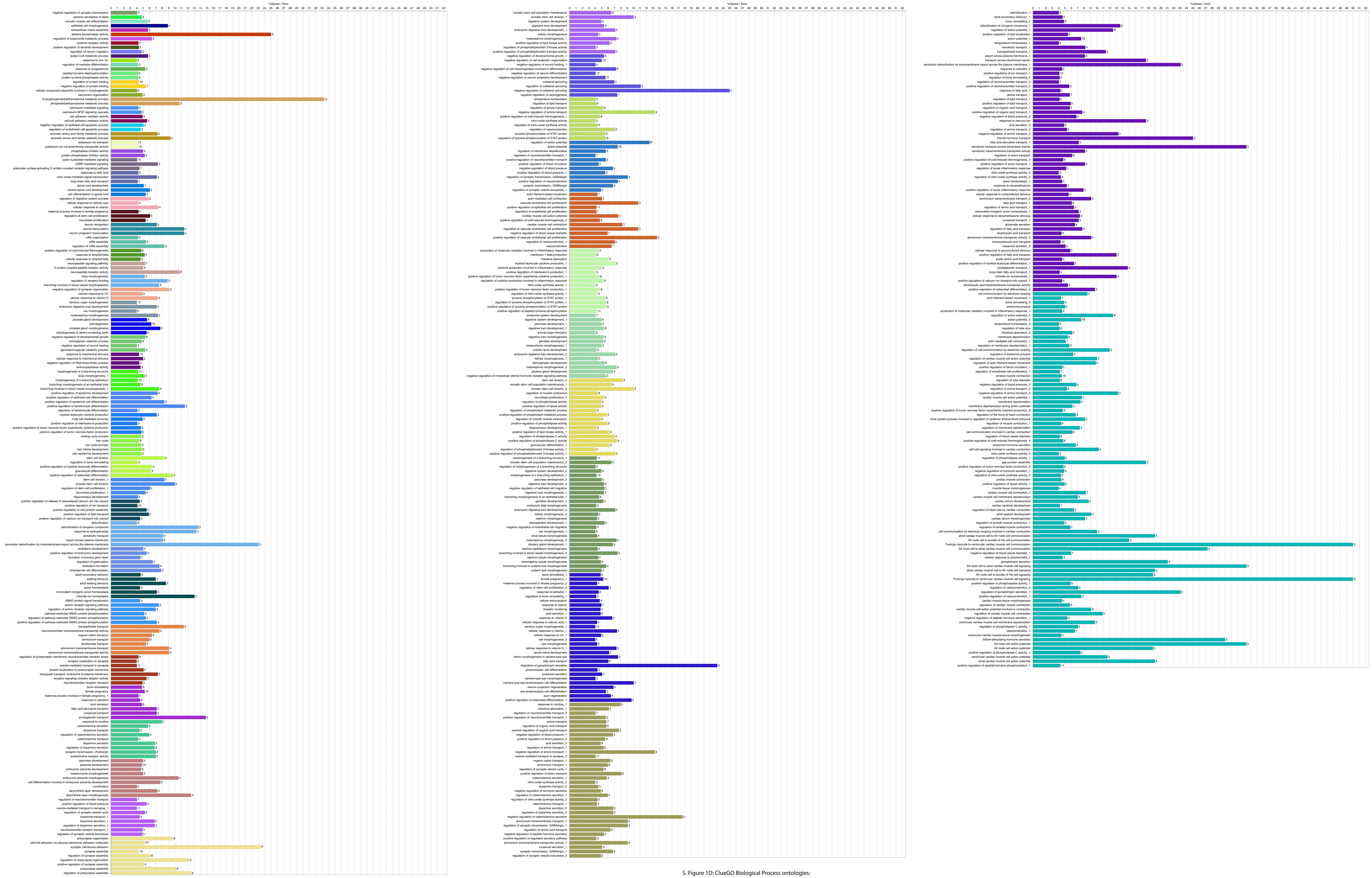

S. Figure 1D: ClueGO Biological Process ontologies:
Number and percent genes per ontology

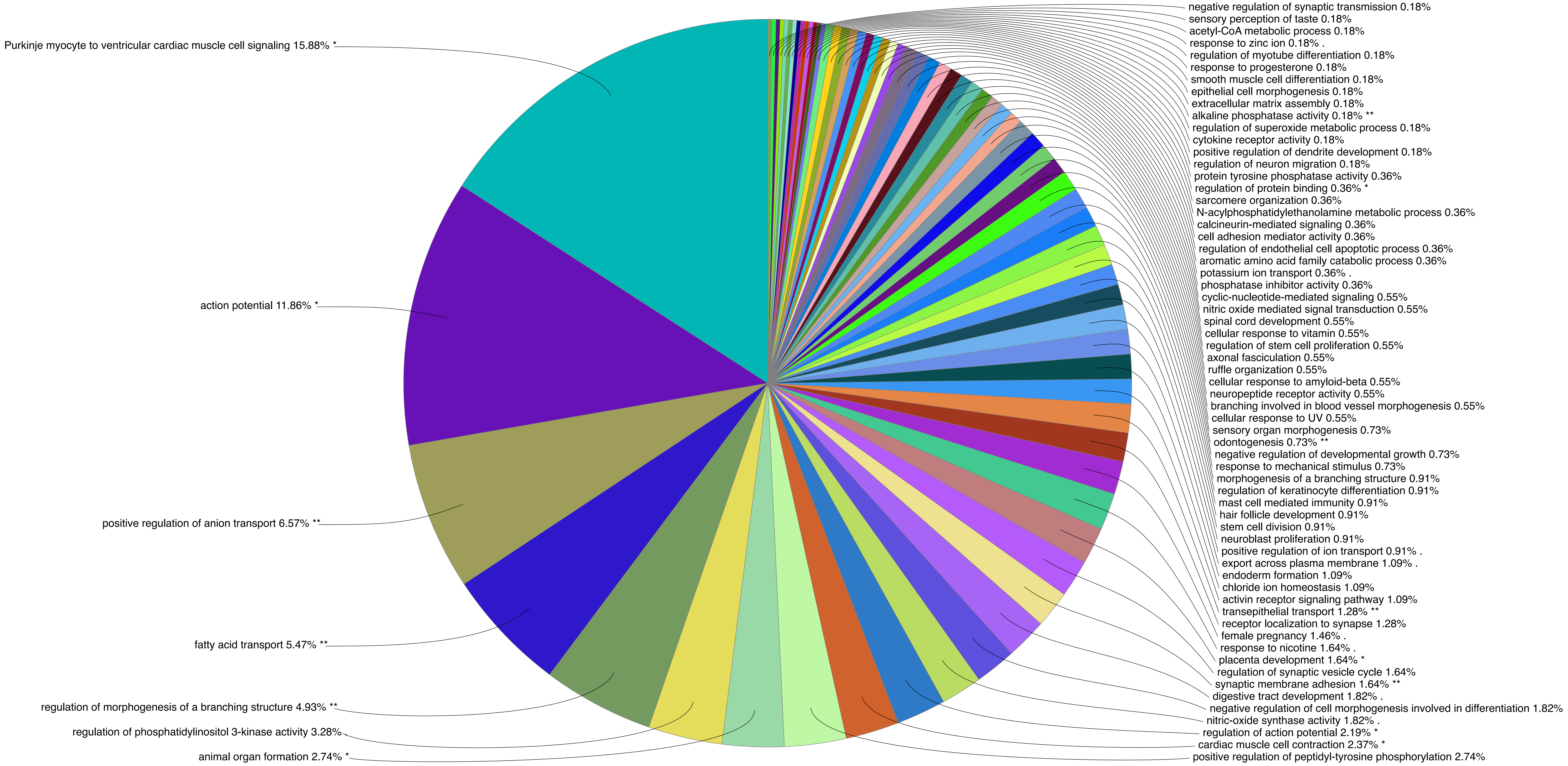

S. Figure 1E: ClueGO Biological Process ontologies, percent representation:
Colors represent ontology groups.

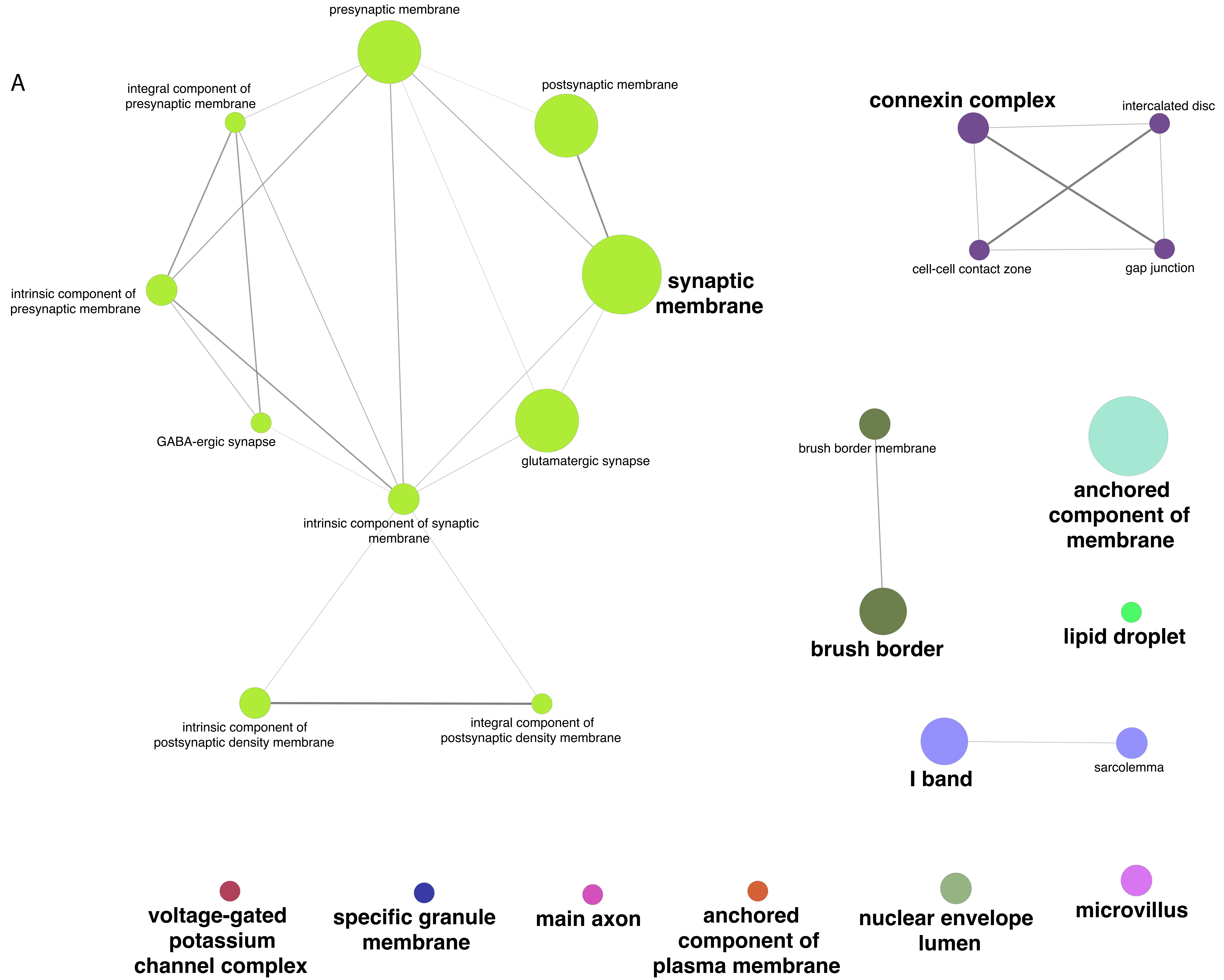

S. Figure 2A: ClueGO Cellular Component network map. Nodes are colored based on ontology and node size indicates significance (all P values < 0.05).

B

crGART to HeLa cellular component %genes per term

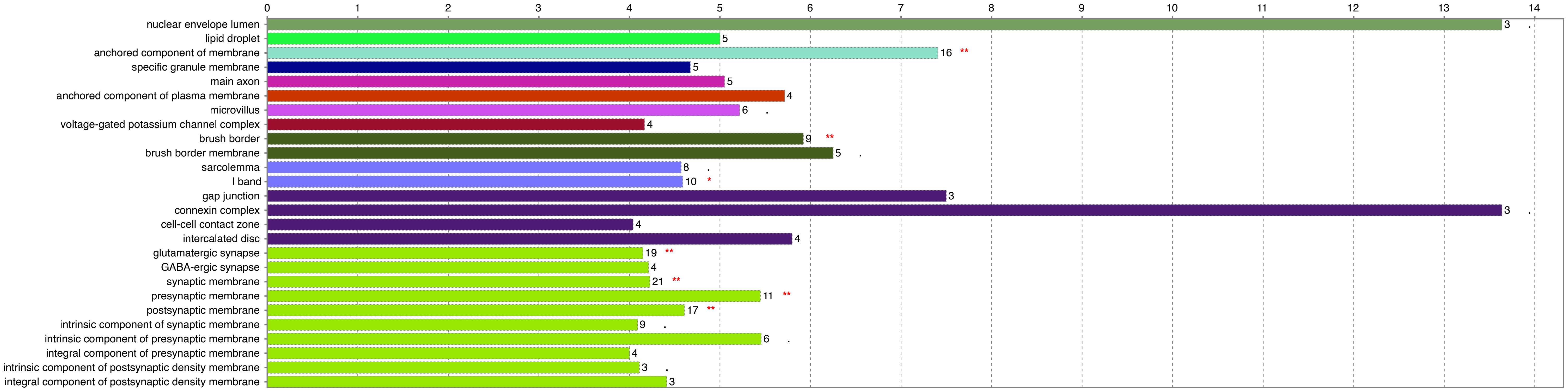

C

crGART to HeLa cellular component terms per group

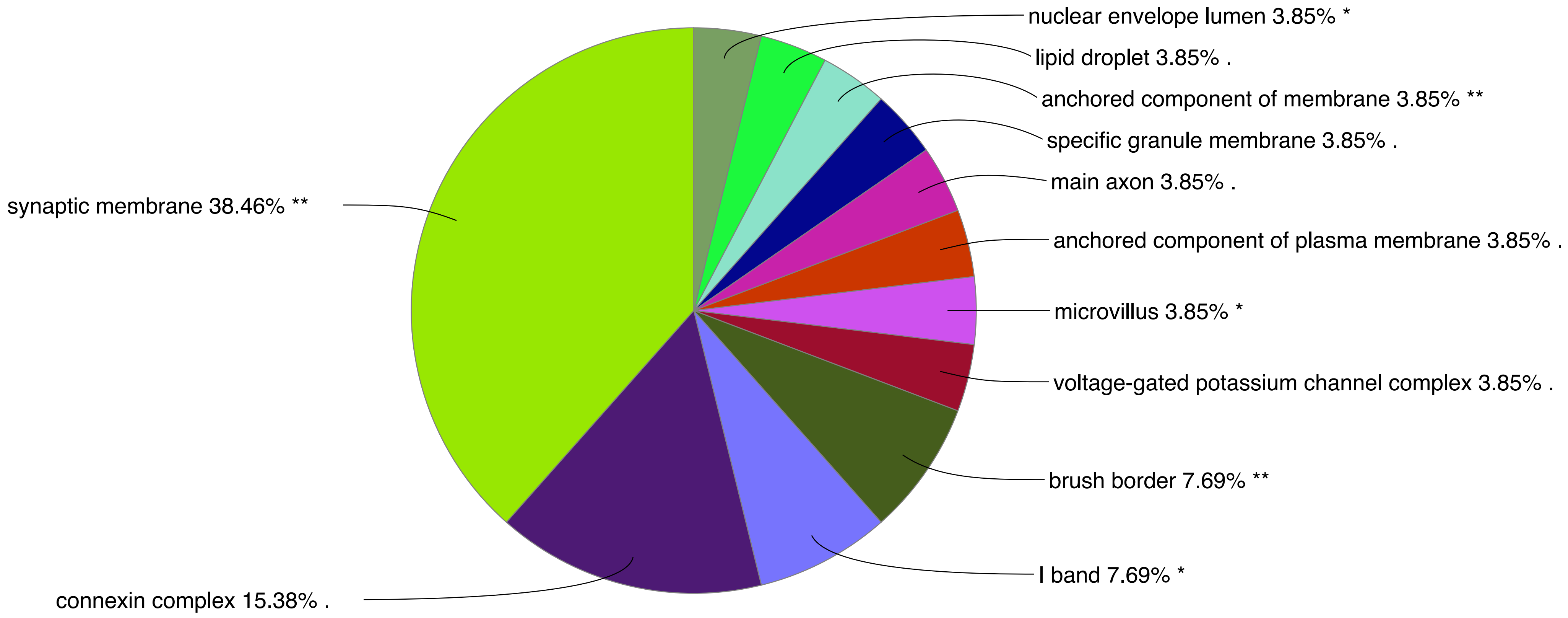

S. Figure 2: B) ClueGO Cellular Component ontologies: Number and percent genes per ontology
C) Percent representation: Colors represent ontology groups.

A

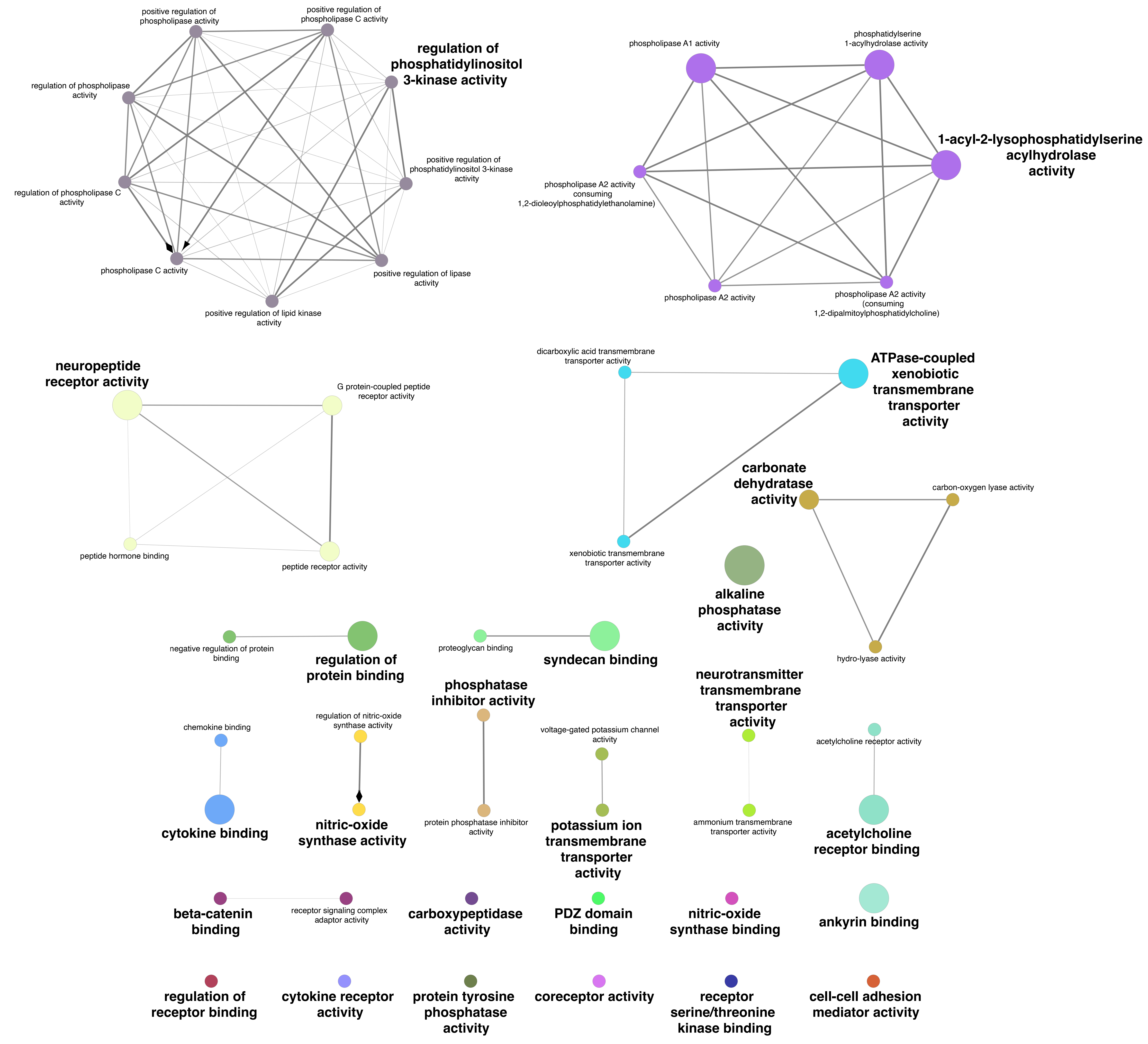

S. Figure 3A: ClueGO Molecular Function network map. Nodes are colored based on ontology and node size indicates significance (all P values < 0.05).

B

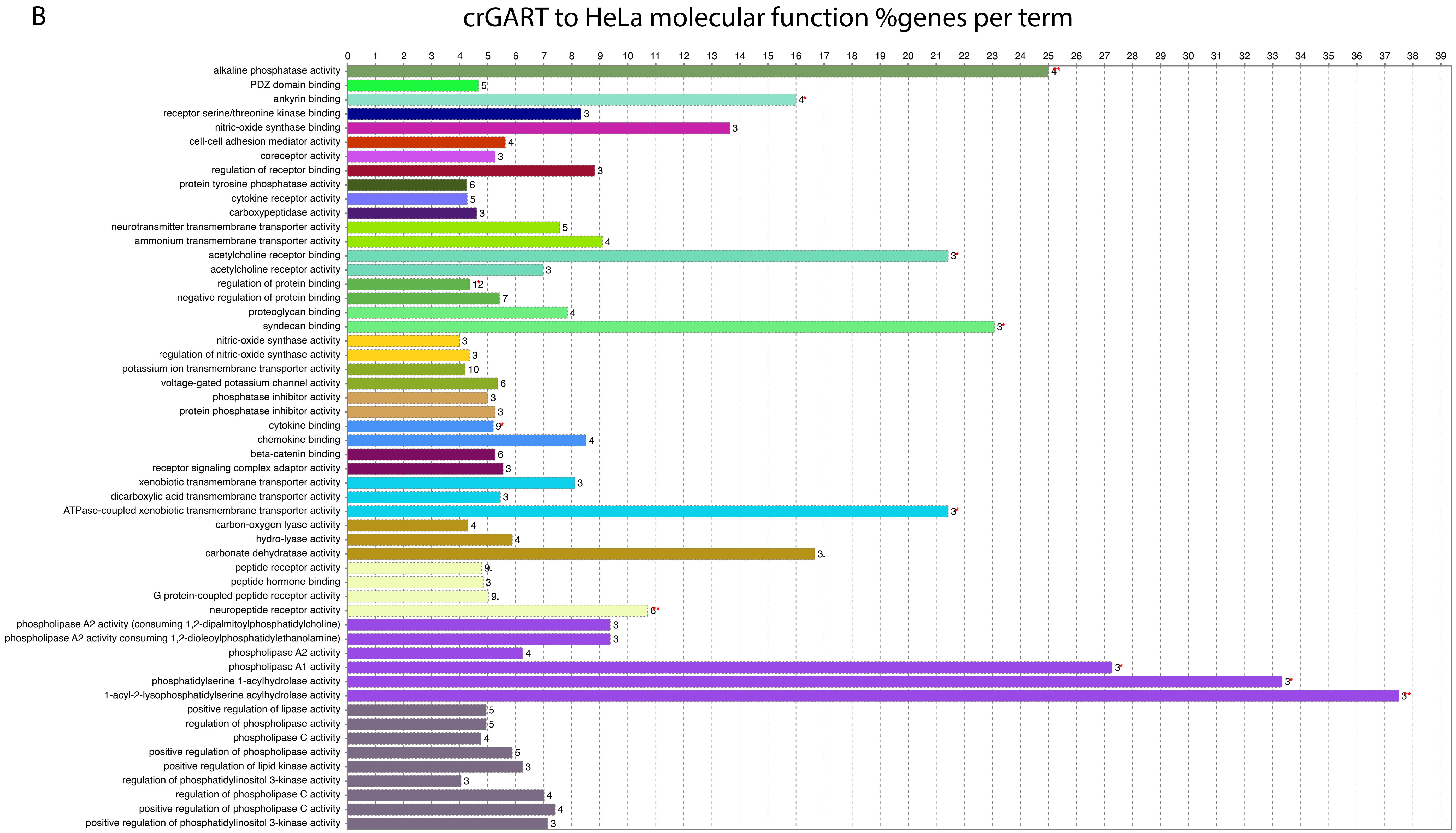

C

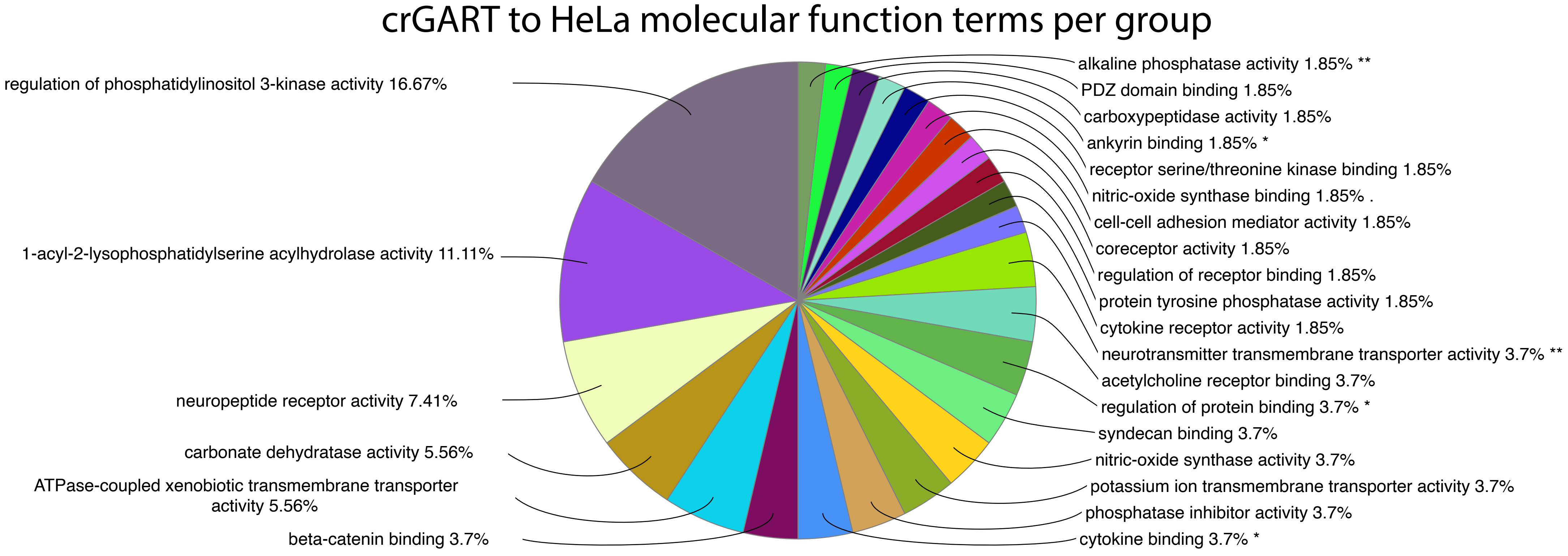

S. Figure 3: B) Molecular Function ontologies: Number and percent genes per ontology
C) Percent representation: Colors represent ontology groups.

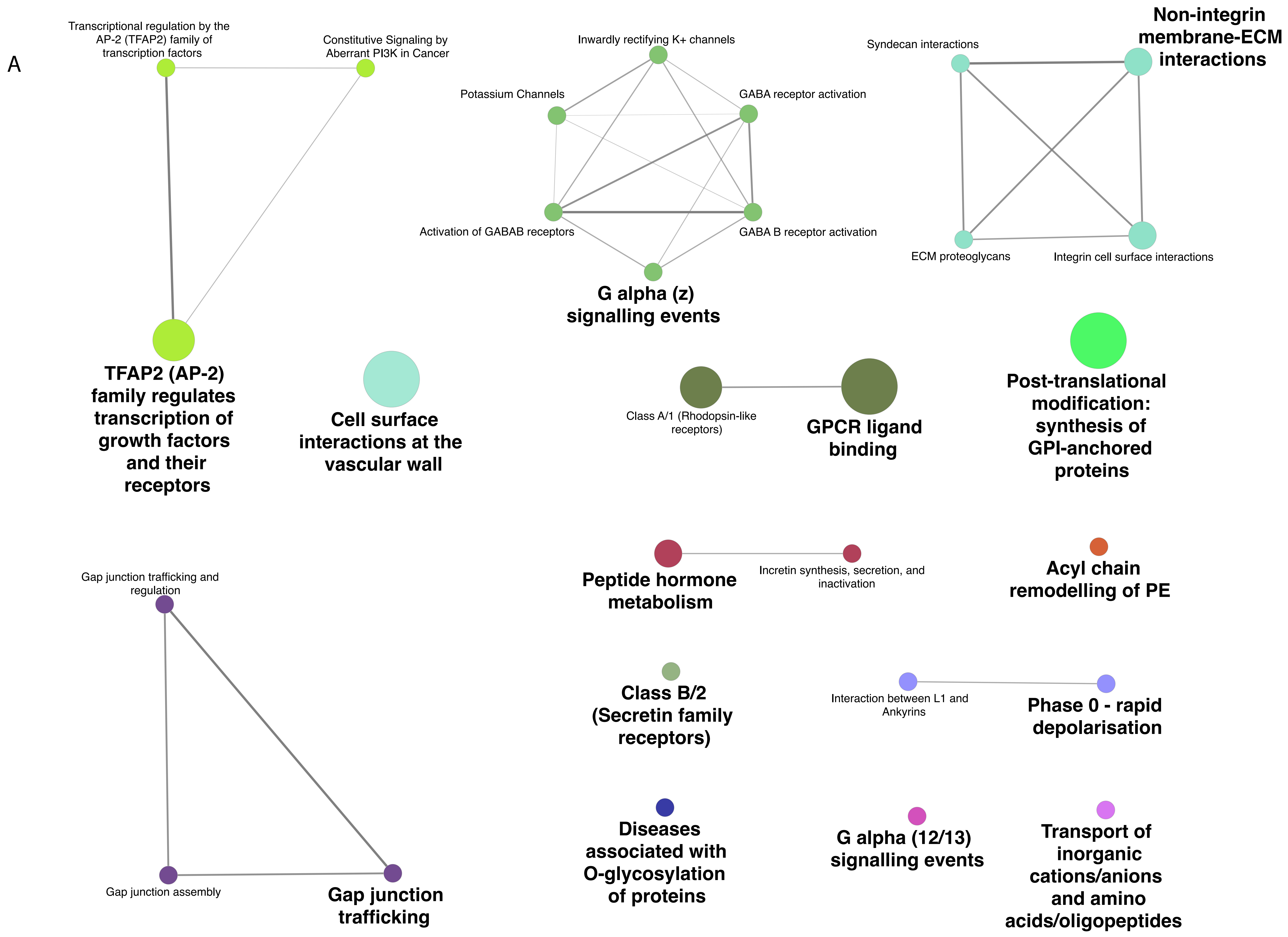

S. Figure 4A: ClueGO Reactome Pathways network map. Nodes are colored based on pathway and node size indicates significance (all P values < 0.05).

B

crGART to HeLa Reactome pathways %genes per term

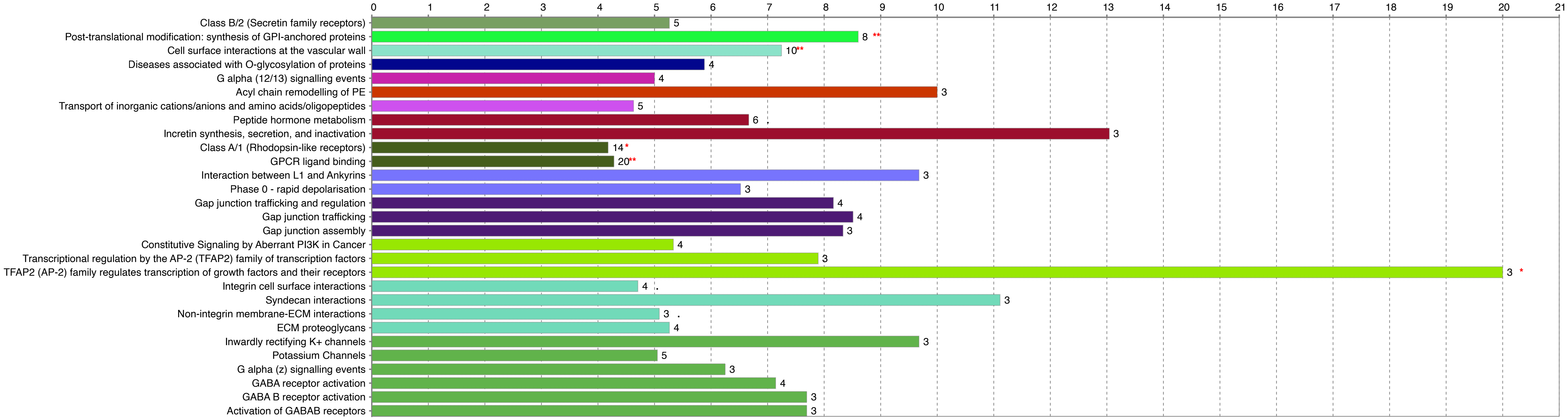

C

crGART to HeLa Reactome pathways terms per group

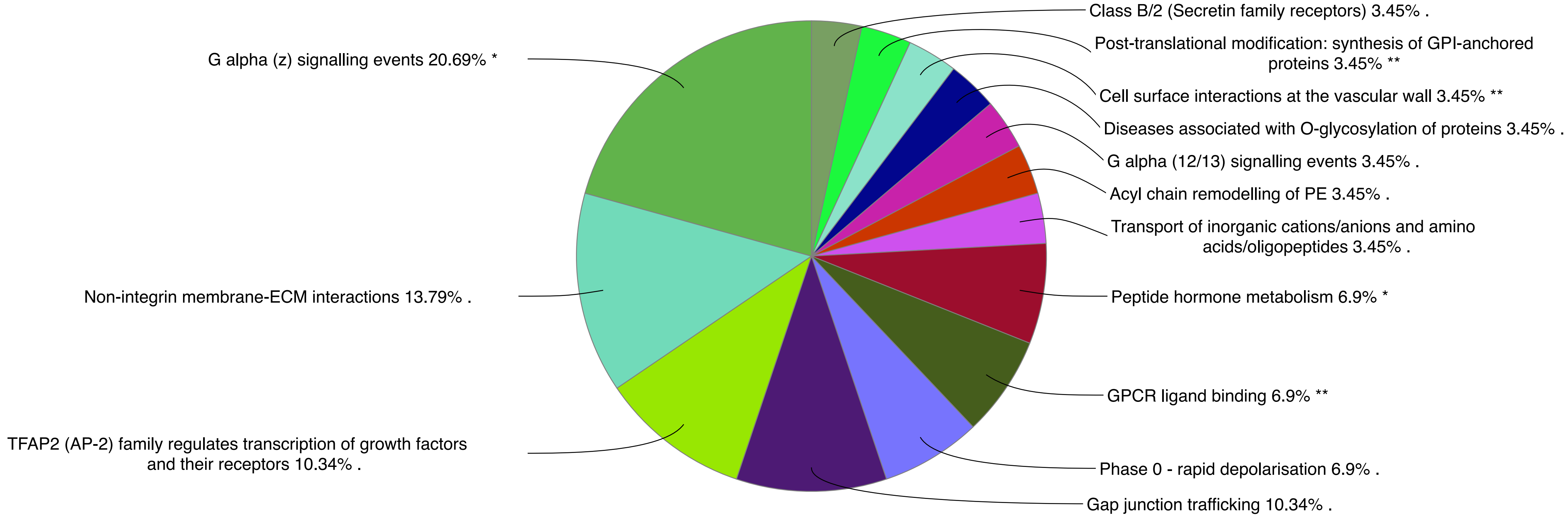

S. Figure 4: B) Reactome Pathways: Number and percent genes per pathway C) Percent representation: Colors represent pathway groups.

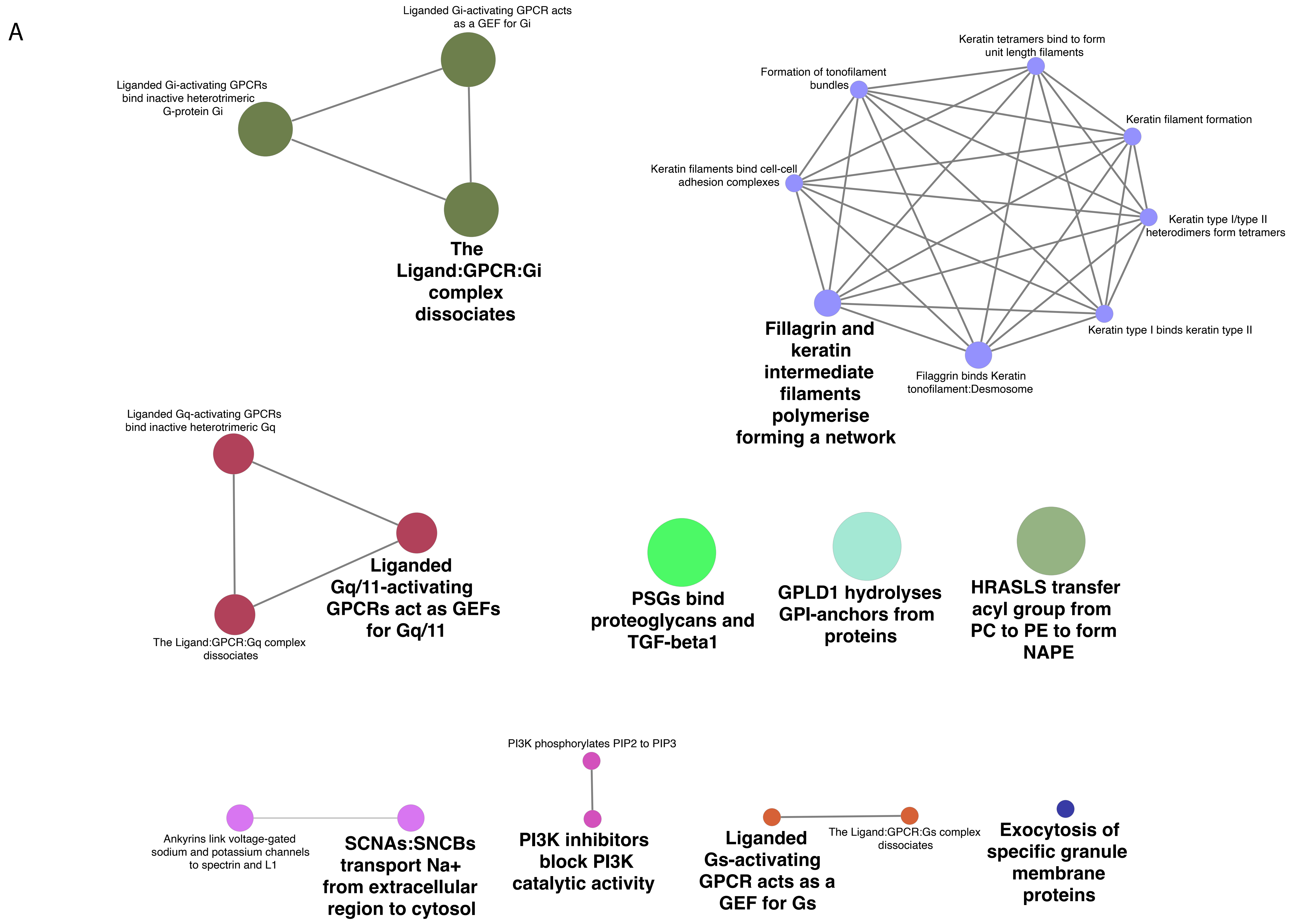

S. Figure 5A: ClueGO Reactome Reactions network map. Nodes are colored based on reaction and node size indicates significance (all P values < 0.05).

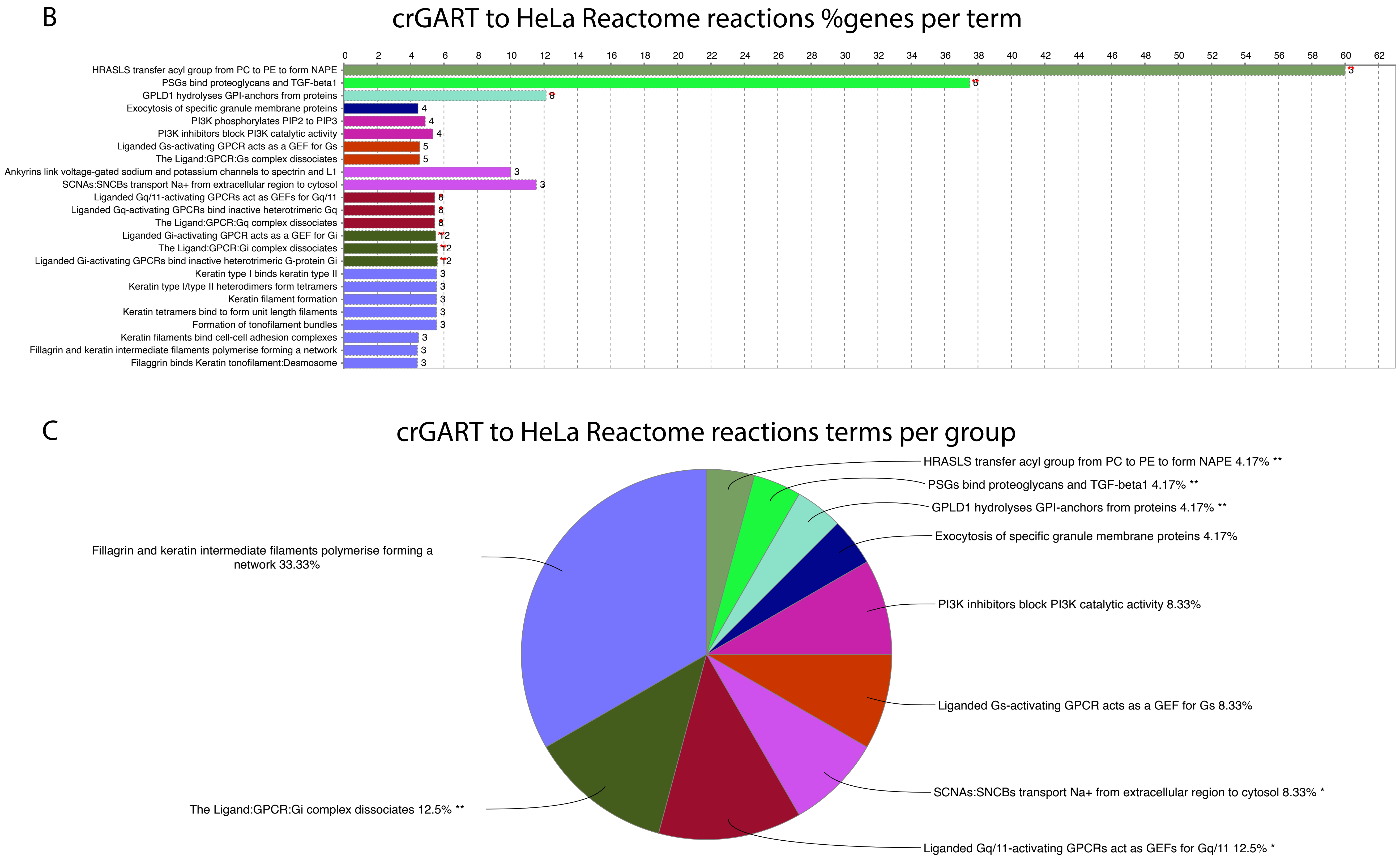

S. Figure 5: B) Reactome Reactions: Number and percent genes per reaction C) Percent representation: Colors represent reaction groups.

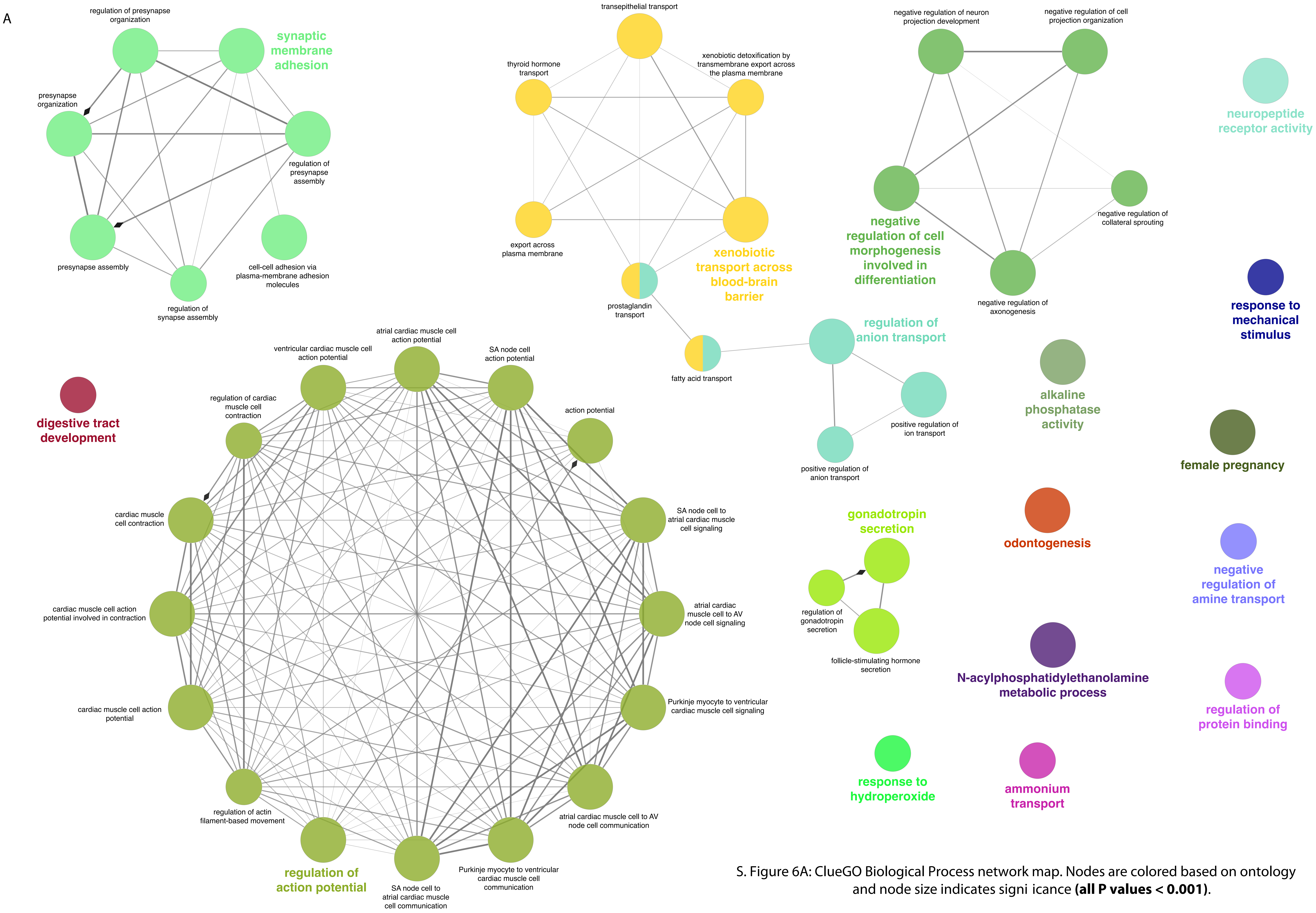

S. Figure 6A: ClueGO Biological Process network map. Nodes are colored based on ontology and node size indicates signi icance **(all P values < 0.001)**.

B

crGART to HeLa biological process %genes per term

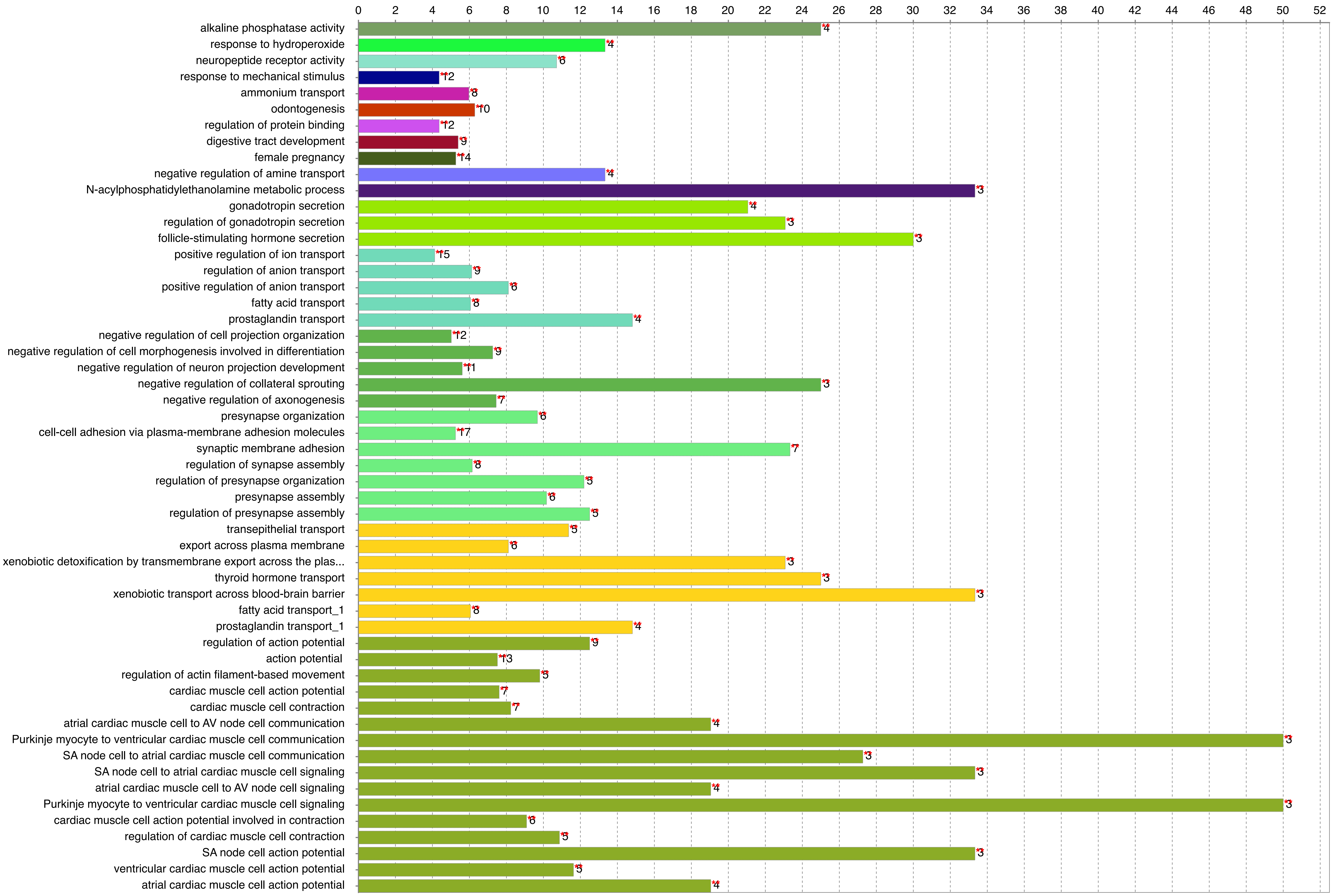

C

crGART to HeLa biological process terms per group

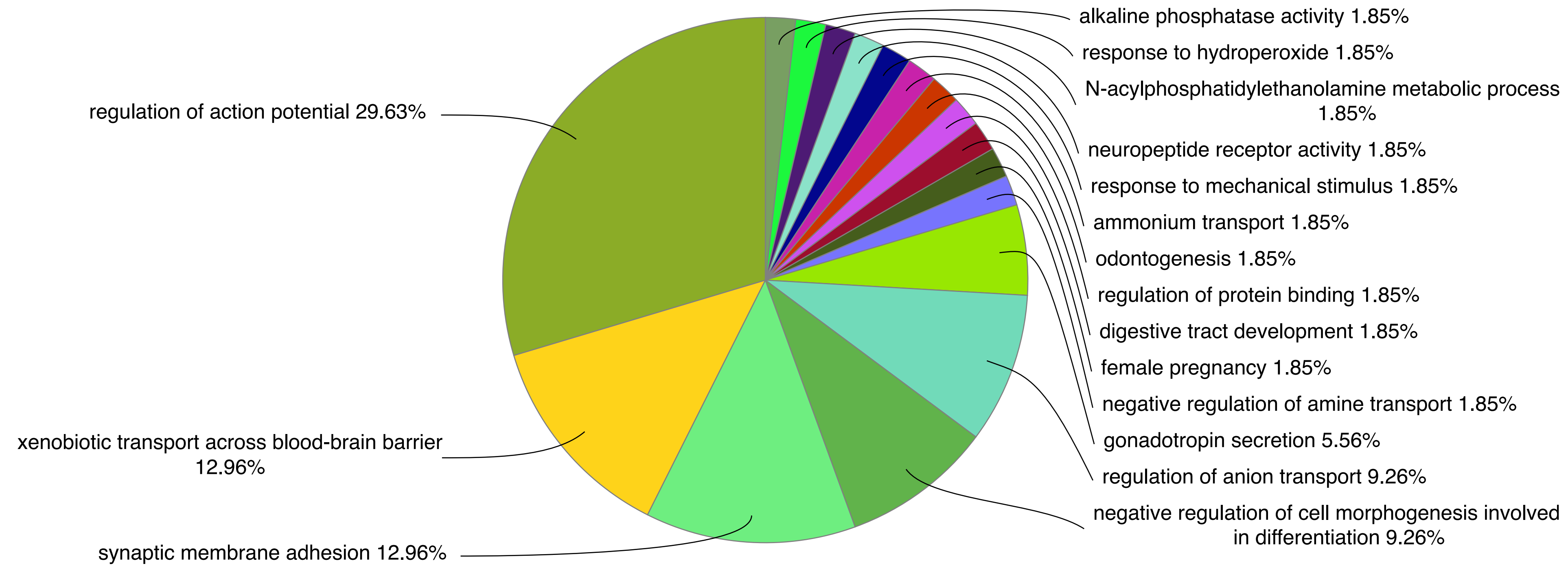

S. Figure 6: B) ClueGO Biological Process ontologies: Number and percent genes per ontology
C) Percent representation: Colors represent ontology groups.
