# Supplementary material for: Transcriptome and metabolome analysis of crGART, a novel cell model of de novo purine synthesis deficiency: Alterations in CD36 expression and activity
Source: PLoS One. 2021 Jul 20;16(7):e0247227. doi: 10.1371/journal.pone.0247227 (PMC8291708; doi:10.1371/journal.pone.0247227)
Supplement: S1 Raw images — (PDF) [file pone.0247227.s008.pdf]

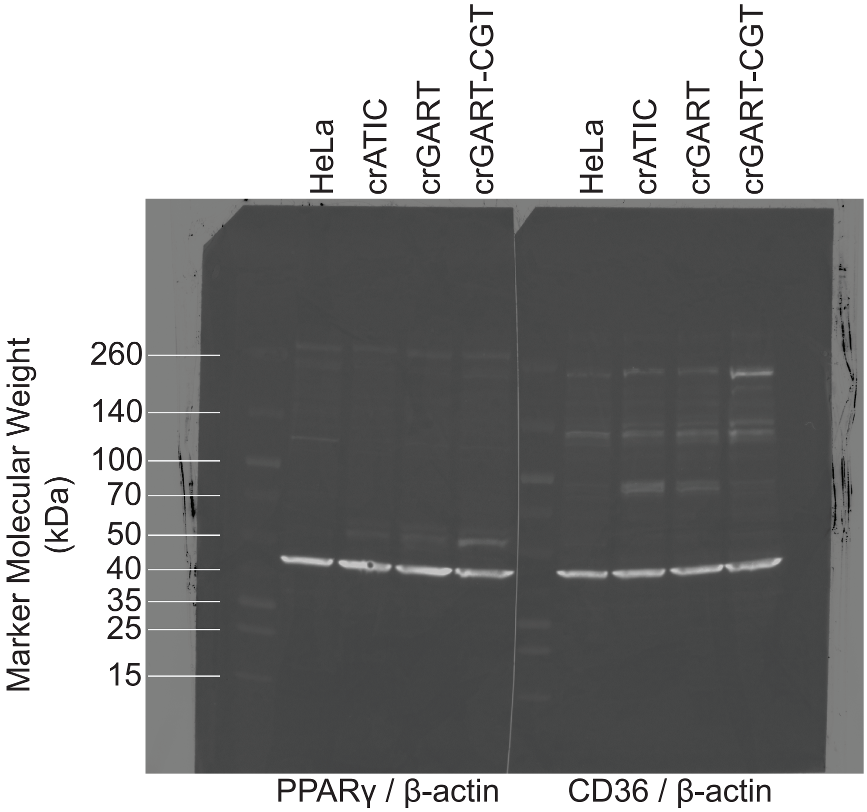

Raw Images: Figure 5B and 5D: The top image is a composite of colorimetric (molecular weight standards) and chemiluminescence. The bottom image shows only chemiluminescence. Images were captured using a ChemiDoc Imaging System (Bio-Rad) following the manufacturer's recommendations for chemiluminescence, 4x4 (default) binning and Image Lab software (Bio-Rad). These images have not been altered or adjusted.

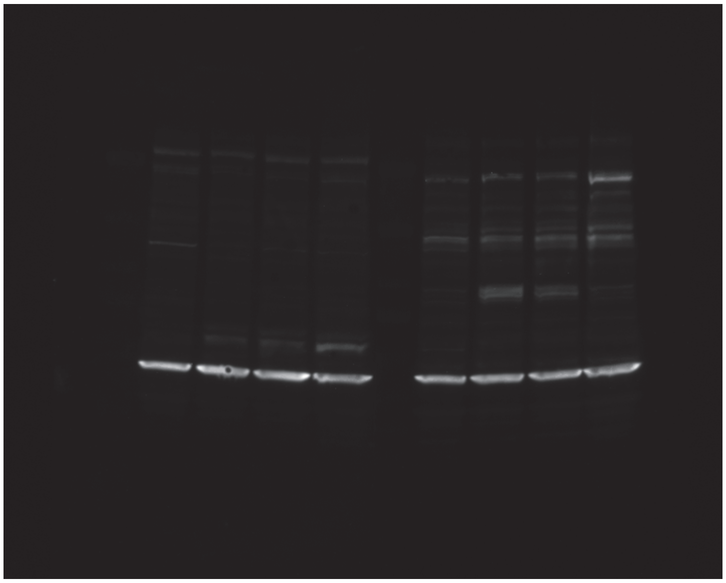

Primary Ab.	Manufacturer	Cat. Number	Dilution
CD36	Abcam	ab133625	1:500
PPARγ	Cell Signaling	2430	1:250
β-actin	Cell Signaling	8457	1:1000
